# Taxonomic novelties in *Scapania*

**DOI:** 10.3897/phytokeys.10.2654

**Published:** 2012-03-12

**Authors:** Jiří Váňa, Jörn Hentschel, Jochen Müller, Jochen Heinrichs

**Affiliations:** 1 Department of Botany, Charles University, Benátská 2, 128 01 Praha 2, Czech Republic; 2 Department of Systematic Botany with Herbarium Haussknecht and Botanical Garden, Friedrich Schiller University, Fürstengraben 1, 07743 Jena, Germany; 3 Department of Systematic Botany, Albrecht von Haller Institute of Plant Sciences, Georg August University, Untere Karspüle 2, 37073 Göttingen, Germany

**Keywords:** Scapaniaceae, nomenclature

## Abstract

Five new supraspecific taxa of Scapania are proposed, *Scapania* subg. *Gracilidae*, *Scapania* subg. *Pseudomacrodiplophyllum*, *Scapania* sect. *Americanae*, *Scapania* sect. *Hyperboreae*, and *Scapania* sect. *Simmonsia*.

## Introduction

**Figure 1.**
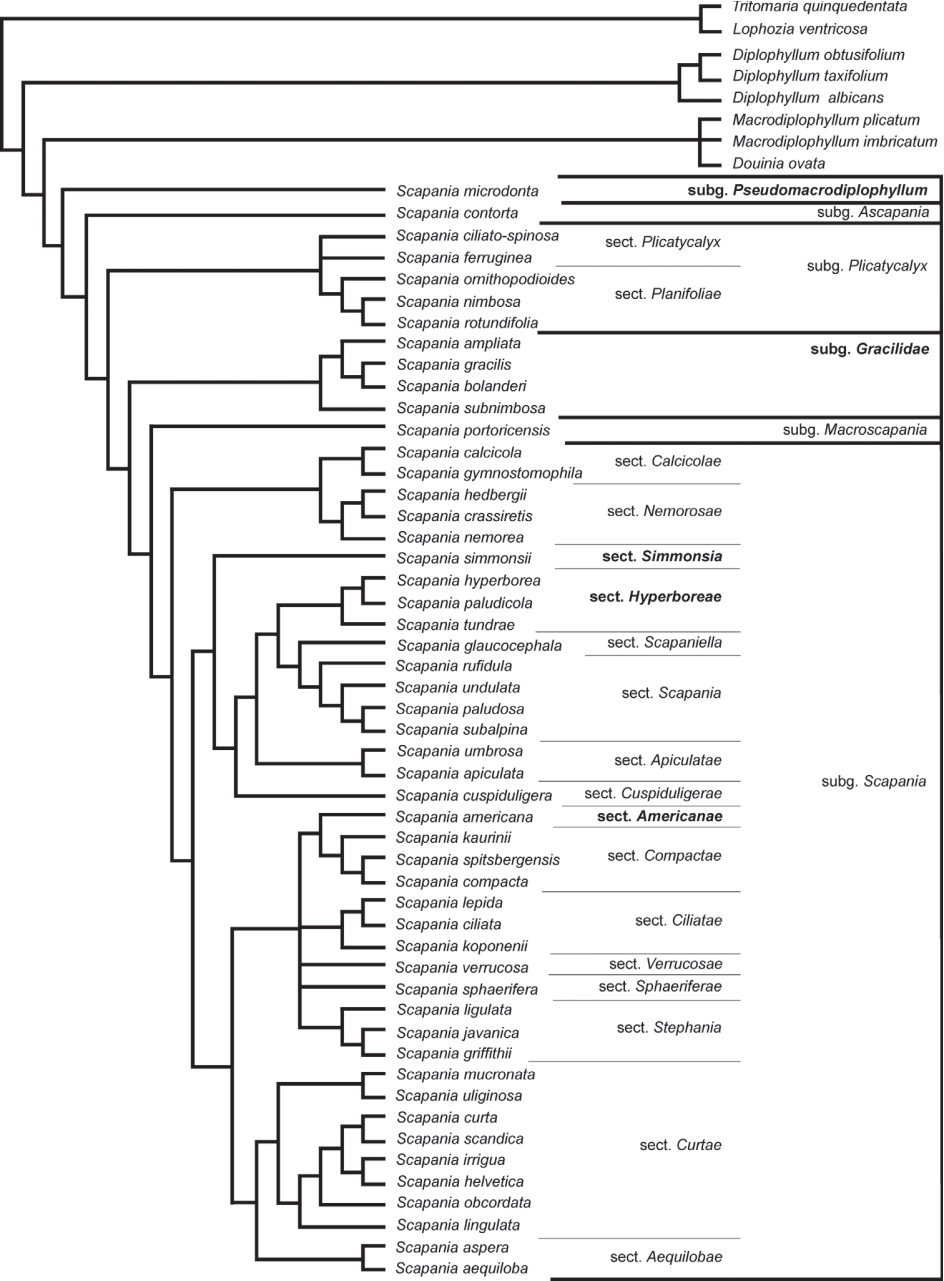
Phylogeny of *Scapania* with the new taxa indicated in bold. Modified from Heinrichs et al. (2012, Fig. 1).

The northern temperate leafy liverwort genus *Scapania* (Dumort.) Dumort. was the subject of two comprehensive molecular phylogenetic studies (Vilnet et al. 2010, Heinrichs et al. 2012). These studies provided evidence for incongruence of the present supraspecific classification (Potemkin 2002) with the molecular tree topologies. Extensive morphological homoplasy hampers a morphological circumscription of several *Scapania* lineages identified in the molecular studies; however, several new combinations and taxa are needed to arrive at a monophyletic supraspecific classification (Fig. 1). In the following, the new taxa are introduced.

## Taxonomy

### Scapania subg. Gracilidae

(H. Buch) Váňa, Hentschel, Joch. Müll. & Heinrichs comb. et stat. nov.

#### Basionym.

*Scapania* sect. *Gracilidae* H. Buch, Commentat. Biol. 3(1): 106. 1928.

#### Type.

*Scapania gracilis* Lindb., Morgonbladet (Helsinki) 1873(286): 2. 1873.

#### Note.

According to the molecular phylogenies presented by Heinrichs et al. (2012), *Scapania* sect. *Gracilidae* forms a lineage outside *Scapania* subg. *Scapania*; hence, we propose subgenus rank (Fig. 1).

### subg. Scapania Pseudomacrodiplophyllum

Váňa, Hentschel, Joch. Müll. & Heinrichs subg. nov.

#### Diagnosis.

*Scapania* subg. *Pseudomacrodiplophyllum* includes species which differ from other *Scapania* elements by the presence of multicellular gemmae with intersecting walls, basal leaf cell walls with intermediate thickenings and pluriplicate perianths.

#### Type.

*Scapania microdonta* (Mitt.) Müll. Frib., Nova Acta Acad. Caes. Leop.-Carol. German. Nat. Cur. 83: 262. 1905.

#### Note.

*Scapania microdonta* [*Macrodiplophyllum microdontum* (Mitt.) Perss.] forms the sister clade to the rest of *Scapania* (Vilnet et al. 2010, Heinrichs et al. 2012); hence, a new subgenus is introduced here to accommodate this species (Fig. 1).

### Scapania sect. Americanae

Váňa, Hentschel, Joch. Müll. & Heinrichs sect. nov.

#### Diagnosis.

The new section of *Scapania* subg. *Scapania* is characterized by its type, the dioicous *Scapania americana* Müll. Frib. Distinctive features of *Scapania americana* and *Scapania* sect. *Americanae* are thick-walled, pigmented stem epidermis cells with thinner-walled cells on the ventral side of the stem, closely and sharply ciliate-dentate leaf-margins, with largest teeth up to five cells long and terminal cells considerably longer than wide, leaf-lobes that are usually decurrent beyond the level of the keel, a verruculose or striate-verruculose cuticle, and two-celled gemmae at the margins of unmodified leaves. The dorsal leaf-lobes often develop an undulate or folded base and may even show minute lobules or auricles.

#### Type.

*Scapania americana* Müll. Frib., Bull. Herb. Boissier, sér. 2, 3: 44. 1902.

#### Note.

*Scapania americana* is placed sister to *Scapania* sect. *Compactae* (Müll. Frib.) H. Buch with weak bootstrap support (Heinrichs et al. 2012). It differs from members of *Scapania* sect. *Compactae* by its dioicous condition; hence we place it in a new section rather than in sect. *Compactae*. In our current circumscription, *Scapania* sect. *Americanae* is monospecific, however, extension of the taxon sampling in forthcoming molecular studies may disclose further representatives.

### Scapania sect. Hyperboreae

Váňa, Hentschel, Joch. Müll. & Heinrichs sect. nov.

#### Diagnosis.

Species of *Scapania* sect. *Hyperboreae* resemble members of *Scapania* sect. *Curtae* (Müll. Frib.) H. Buch but differ by the presence of brownish to reddish gemmae, and a usually larger size.

#### Type.

*Scapania hyperborea* Jørg., Förh. Vidensk.-Selsk. Kristiania 1894(8): 56. 1894.

#### Note.

The presence of the type species of *Scapania* sect. *Irriguae* (Müll. Frib.) H. Buch, *Scapania irrigua* (Nees) Nees, in *Scapania* sect. *Curtae* necessitates the introduction of a new section for the remaining elements of *Scapania* sect. *Irriguae*. Molecular data so far supported the presence of *Scapania paludicola* Loeske & Müll. Frib. and *Scapania tundrae* (Arnell) H. Buch in *S.* subg. *Scapania* sect. *Hyperboreae*, as well as *Scapania hyperborea* (Vilnet et al. 2010; Heinrichs et al. 2012).

### Scapania sect. Simmonsia

(R.M. Schust.) Váňa, Hentschel, Joch. Müll. & Heinrichs comb. et stat. nov.

#### Basionym.

*Scapania* subsect. *Simmonsia* [“*Simmonsiae*”] R.M. Schust., Hepat. Anthocerotae N. Amer. 3: 612. 1974.

#### Type.

*Scapania simmonsii* Bryhn & Kaal., Rep. Second Norweg. Arctic Exped. Fram 2 (11): 51. 1906.

#### Note.

*Scapania simmonsii* forms an isolated lineage within *S.* subg. *Scapania*, hence, we erect a section for this species rather than using subsectional rank (Schuster 1974). Distinctive features of the monotypic section are the deflexed-involute, broad, concave ventral leaf lobes that stand away from the stem at almost right angles and leaf cell walls with very large, nodulose trigones.

## Supplementary Material

XML Treatment for Scapania subg. Gracilidae

XML Treatment for Scapania Pseudomacrodiplophyllum

XML Treatment for Scapania sect. Americanae

XML Treatment for Scapania sect. Hyperboreae

XML Treatment for Scapania sect. Simmonsia

## References

[B1] Buch H (1928) Die Scapanien Nordeuropas und Sibiriens – 2. Systematischer Teil. Commentationes Biologicae 3 (1): 1-177

[B2] Heinrichs J Bombosch A Feldberg K Kreier HP Hentschel J Eckstein J Long D Zhu RL Schäfer-Verwimp A Schmidt AR Shaw B Shaw AJ Váňa J (2012) A phylogeny of the northern temperate leafy liverwort genus *Scapania* (Scapaniaceae, Jungermanniales). Molecular Phylogenetics and Evolution 62: 973-985 doi: 10.1016/j.ympev.2011.11.029 22155360 10.1016/j.ympev.2011.11.029

[B3] Potemkin AD (2002) Phylogenetic system and classification of the family Scapaniaceae Mig. emend. Potemkin (Hepaticae). Annales Botanici Fennici 39 (4): 309-334

[B4] Schuster RM (1974) The Hepaticae and Anthocerotae of North America. Columbia University Press, New York.

[B5] Vilnet AA Konstantinova NA Troitsky AV (2010) Molecular insight on phylogeny and systematics of Lophoziaceae, Scapaniaceae, Gymnomitriaceae and Jungermanniaceae. Arctoa 19: 31-50

